# Association of lipid accumulation product with chronic kidney disease in Chinese community adults: a report from the REACTION study

**DOI:** 10.1186/s12944-021-01569-8

**Published:** 2021-10-09

**Authors:** Pijun Yan, Yong Xu, Ying Miao, Qian Tang, Yuru Wu, Xue Bai, Zhihong Zhang, Qian Li, Qin Wan

**Affiliations:** 1 Department of Endocrinology, the Affiliated Hospital of Southwest Medical University, Luzhou, 646000 Sichuan China; 2 Department of General Medicine, the Affiliated Hospital of Southwest Medical University, Luzhou, 646000 Sichuan China; 3 Southwest Medical University, Luzhou, 646000 Sichuan China

**Keywords:** Lipid accumulation product, Chronic kidney disease, Visceral obesity, Albuminuria, Estimated glomerular filtration rate, Chinese community adults, Stratified analysis

## Abstract

**Background:**

Limited studies regarding the correlation of lipid accumulation product (LAP) with a decreased estimated glomerular filtration rate (eGFR) have yielded conflicting findings, and no report has demonstrated the relationship of LAP with chronic kidney disease (CKD), defined as the presence of albuminuria and/or a decreased eGFR. The purpose of this study was to estimate the possible correlation of LAP with CKD prevalence in Chinese community adults.

**Method:**

In this cross-sectional study, LAP level of 7202 participants (age ≥ 40 years) was determined, and its possible association with CKD was evaluated by a multiple logistic regression model.

**Results:**

Compared with subjects with non-CKD, non-albuminuria, and high eGFR, LAP levels significantly increased in female not male subjects with CKD, albuminuria, and low eGFR, respectively (all *P* < 0.001). The univariate logistic regression analysis revealed that LAP level of female not male subjects were significantly and positively associated with the prevalence of CKD (*P* < 0.001). The multivariate logistic regression analysis showed that the risk of CKD prevalence in female not male subjects progressively increased across LAP quartiles (P for trend < 0.01), and the risk of CKD prevalence of subjects in Q4 significantly increased compared to those in Q1 after adjustment for potential confounding factors in Models 4 (odds ratio [OR]: 1.382, 95% confidence intervals [CI] 1.002–1.906, *P* < 0.05). Stratified analysis revealed positive associations of LAP quartiles with risk of CKD prevalence in people with the following characteristics: women, older, overweight, with hypertension, normal glucose tolerance, appropriate low-density lipoprotein cholesterol, nonsmokers, nondrinkers, and no cardiovascular disease events.

**Conclusions:**

High LAP levels might be significantly associated with risk of CKD prevalence in community-dwelling Chinese female adults, which may inform both public health recommendations and clinical practice.

## Background

Chronic kidney disease (CKD), a chronic condition presented as kidney structural or functional abnormalities caused by multiple factors, has been reported to be a global public health issue with an increasing incidence worldwide [1]. It is estimated that CKD impacts ~ 10% of the world’s population [2] and 11.6% of the Chinese adult population in 2018 [3]. Emerging studies have shown that CKD is related to an increased risk of unfavourable health outcomes, such as cognitive impairment, end-stage renal disease, and atherosclerotic cardiovascular disease (CVD) morbidity and mortality [2, 4, 5]. However, CKD is initially often silent without warning, and a considerable proportion of patients have already developed CVD when CKD is diagnosed. Therefore, the availability of a simple, noninvasive and reliable index for the early identification of individuals who were at high risk for CKD and further determination of effective strategies to prevent its development and progression are of great significance.

Compelling data indicate that obesity, especially central obesity, is involved in the development and progression of CKD and its components, including a high urinary albumin–creatinine ratio (ACR) of ≥30 mg/g, also called albuminuria, and a low estimated glomerular filtration rate (eGFR) of < 60 mL/min/1.73 m2 [5–8], but the mechanisms underlying the associations are multifactorial and still unclear. It has been well established that visceral adipose tissue (VAT) can secrete several proinflammatory cytokines and adipocytokines that lead to inflammation, oxidative stress, insulin resistance, and endothelial dysfunction and subsequently induce kidney structure and function changes such as glomerular sclerosis and albuminuria, ultimately resulting in renal dysfunction [6, 9–11]; thus, the cardiometabolic risks of VAT are higher than subcutaneous adipose tissue (SAT) [6]. In routine clinical practice, the magnetic resonance imaging (MRI) and/or computed tomography (CT), which are considered the gold standard to directly measure VAT, are not feasible because these techniques are time-consuming, costly, and radioactive [9]. Waist circumference (WC), a practical anthropometric parameter to identify central obesity, is unable to distinguish between VAT and SAT [6, 9]. In recent years, the lipid accumulation product (LAP), calculated as a combination of fasting triglycerides (TG) and WC, has been considered as a novel surrogate marker of central adiposity and alternative continuous index of central lipid accumulation [11, 12]. Given the close associations between central adiposity and CKD, it is plausible that LAP might be significantly associated with CKD. However, limited studies regarding the relationship between LAP and low eGFR in specific targeted populations have yielded conflicting findings [11, 13–15]. Several studies showed that LAP was significantly associated with low eGFR in Korean adults, rural populations in Northeast China, and populations in East China [11, 13, 14]; however, other study indicated that LAP quartiles had no significant association with the incidence of low eGFR among Iranian urban residents without diabetes [15]. Moreover, the relationship of LAP with CKD defined as low eGFR and/or albuminuria [3] has not yet been investigated.

Thus, this cross-sectional study, which was developed from the longitudinal Risk Evaluation of Cancers in Chinese Diabetic Individuals (REACTION) study, aimed to explore the correlation of LAP with CKD prevalence in the general population in China.

## Methods

### Study population

**Fig. 1:**
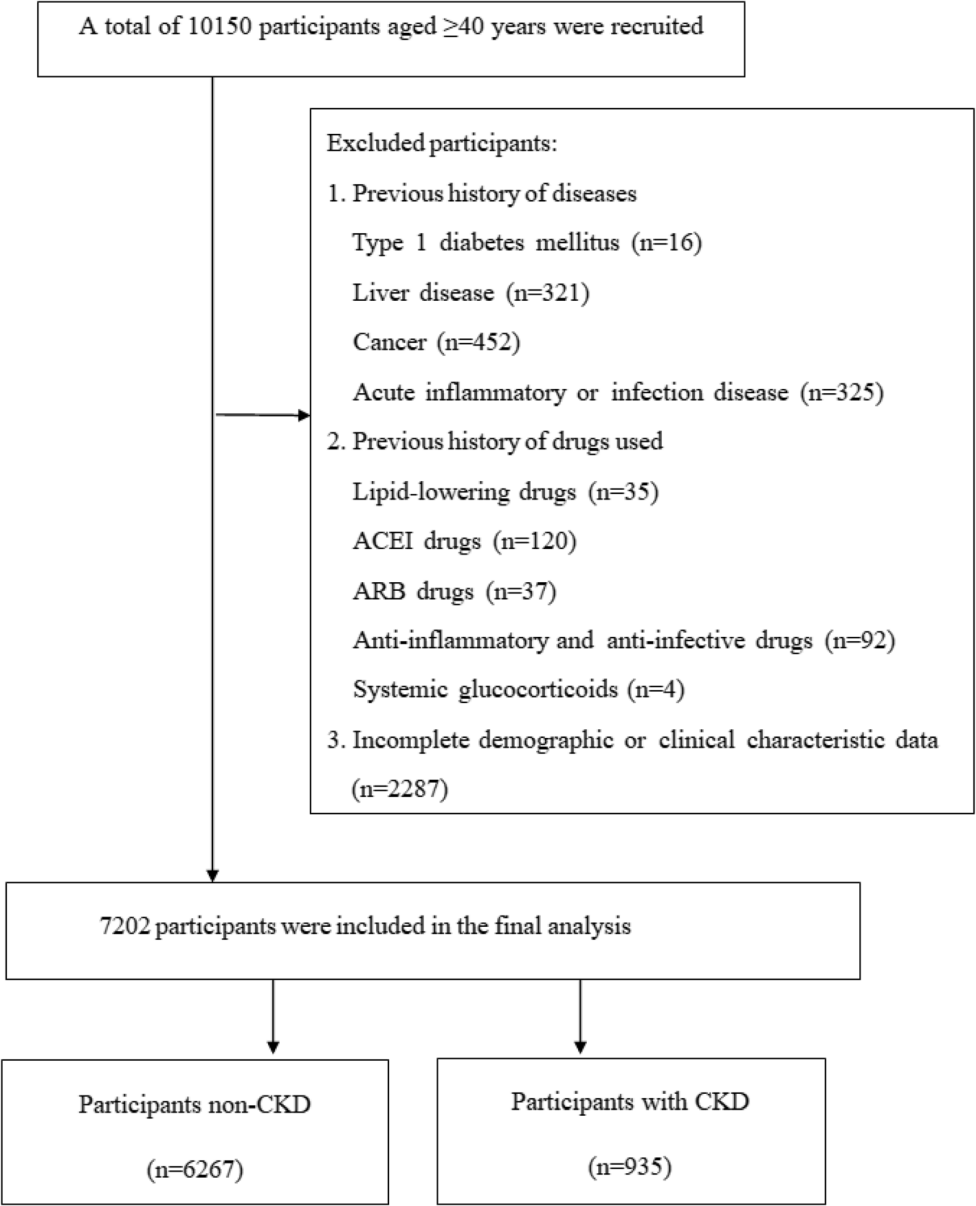
Flow chart of the selection of study participants

This current study was developed from the REACTION study, which is a national, multicentre study led by Rui-Jin Hospital affiliated with Shanghai Jiao-Tong University School of Medicine investigating the correlation of prediabetes and type 2 diabetes mellitus (T2DM) with cancer risk in the Chinese population [16–18]. The present study population was from Luzhou, Sichuan Province. Between May and December 2011, 10,150 participants (age ≥ 40 years) were initially recruited. Participants with type 1 diabetes mellitus, liver disease, cancer, acute inflammatory or infectious disease, those using angiotensin receptor blocker (ARB)/angiotensin-converting enzyme inhibitors (ACEIs) drugs, anti-inflammatory and anti-infective drugs, systemic glucocorticoids, lipid-lowering drugs, and those with incomplete demographic or clinical characteristics data were excluded (Fig. 1). Overall, the final analysis included 7202 subjects.

The present study was conducted in accordance with the guidelines set out in the Declaration of Helsinki, and the Research Ethics Committees of Rui-Jin Hospital affiliated with Jiao-Tong University School of Medicine approved all procedures associated with human participants (clearance no. for ethical approval. 201,114). All participants made an informed written consent to participate.

### Data collection and measurements

An in-person interview and standard questionnaire was used by Trained study personnel in local community clinics to obtain data on participants’ demographic information (age and sex), lifestyle behaviours (smoking status and alcohol intake), family and personal medical history, and medication use. According to cigarette smoking habits, smoking status was classified as never, former and current smokers [18]. The study participants were also categorized as never, former and current drinkers based on the type and frequency of alcohol consumption recorded [17, 18]. Before breakfast, body weight, height and WC of the participants were measured, and then the body mass index (BMI) was obtained based on height and body weight [16, 17]. The LAP index was calculated for women [TG×(WC-58)] and men [TG×(WC-65)], respectively [11, 12]. According to the LAP index, the whole study participants were grouped into quartiles: Q1, LAP < 16.20; Q2, 16.20 ≤ LAP < 28.88; Q3, 28.88 ≤ LAP ≤50.16; Q4, LAP > 50.16. Moreover, the subjects were also grouped into quartiles based on the LAP index: Q1, LAP < 13.69; Q2, 13.69 ≤ LAP < 25.79; Q3, 25.79 ≤ LAP ≤46.77; Q4, LAP > 46.77 for male subjects, and Q1, LAP < 17.43; Q2, 17.43 ≤ LAP < 30.35; Q3, 30.35 ≤ LAP ≤51.48; Q4, LAP > 51.48 for female subjects. Systolic blood pressure (SBP) and diastolic blood pressure (DBP) were measured in all participants in a seated position three times at 5-min intervals, and then the average of the three readings was used.

A 75 g oral glucose tolerance test was performed in the participants after overnight fasting for at least 10 h, and blood samples were obtained by centrifugation at 0 and 2 h and stored at − 80 °C. Blood glucose [glycated haemoglobin A1C (HbA1c), fasting blood glucose (FBG), 2-h postload blood glucose (PBG)], lipid profiles, including low-density and high lipoprotein cholesterol (LDL-C, HDL-C), total cholesterol (TC), TG, and creatinine (Cr) were measured according to relevant protocols and guidelines at a certified central laboratory, which is accredited in accordance with the International Organization for Standardization (ISO)15,189.

Urinary albumin concentration and Cr levels of first morning urine specimens were determined using chemiluminescence immunoassay, and then the urinary ACR (mg/g Cr) was calculated. According to the equation of Japanese coefficient-modified Chronic Kidney Disease Epidemiology Collaboration (CKD-EPI), the eGFR was calculated [2, 11].

### Definition of variables

According to the Working Group for Obesity in China (WGOC) criteria, BMI ≥28 kg/m2 was categorized as obesity, and 24 ≤ BMI < 28 kg/m2 was classified as overweight [19]. According to previously published criteria, the subjects were grouped into three groups: high (LDL-C ≥ 4.1 mmol/L), borderline high (3.4 mmol/L ≤ LDL-C < 4.1 mmol/L), and appropriate (LDL-C < 3.4 mmol/L) [20]. Diagnosis of normal glucose tolerance (NGT), prediabetes, and T2DM was based on previously published criteria [16, 17]. Hypertension and CVD events [coronary heart disease (CHD), stroke, myocardial infarction (MI), and peripheral arterial disease (PAD)] were diagnosed as described previously [11, 16–18, 20, 21].

### Statistical analysis

All data in the present study was analyzed by Statistical Package for Social Sciences (SPSS) version 20.0. According to the distribution of variables, continuous variables are expressed as the mean ± standard deviation (SD) and median (25th, 75th percentile), respectively. Categorical variables are presented as numbers (percentages).

Comparisons between two groups were conducted with χ2 test for categorical variables, Student’s t test or Mann–Whitney U test for continuous variables. The correlation of LAP levels and other variables with the risk of CKD prevalence were determined by univariate logistic regression analyses in men and women, respectively. Then, multivariate-adjusted logistic regression analysis was used to investigate the correlation of LAP quartiles with the risk of CKD prevalence in men and women, respectively. Odds ratio (OR) and corresponding 95% confidence interval (CI) were calculated. Stratified analyses were conducted among different gender, age, BMI, current smoking and drinking status, prevalence of CVD events,glucose metabolism state, blood pressure, and LDL-C subgroups to investigate the relevant factors underlying the correlation of LAP with CKD prevalence. Potentialinteractions of LAP quartiles and strata variables were assessed.

Two-tailed *P* values < 0.05 were considered to have statistical significance.

## Results

### Demographic and clinical characteristics of the study population

**Fig. 2:**
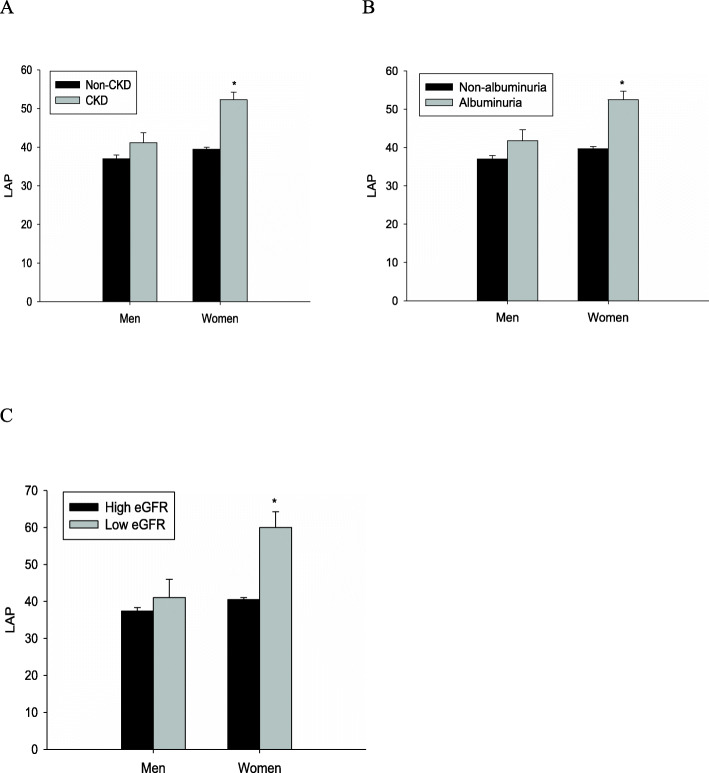
LAP levels in male and female subjects by CKD, albuminuria, and low eGFR, respectively. **A**. LAP levels in male and female subjects by CKD, respectively. **B**. LAP levels in male and female subjects by albuminuria, respectively. C. LAP levels in male and female subjects by low eGFR, respectively. Vs. non-CKD group or non-albuminuria group or high eGFR, *P < 0.001

Table 1 revealed the study population basic characteristics in men and women, respectively. In male subjects, people with CKD had significantly more users of hypoglycaemic drugs, fewer current drinkers, older age, poorer kidney function (elevated serum Cr and urinary ACR, and reduced eGFR), higher SBP, TC, LDL-C, blood glucose (FBG, PBG, and HbA1c), prevalence of hypertension, T2DM, CVD events (CHD and PAD), and lower height compared with those without (*P* < 0.001 or *P* < 0.01 or *P* < 0.05; Table 1 and Fig.2). In female subjects, people with CKD had also significantly more users of hypoglycaemic drugs, older age, poorer kidney function (elevated serum Cr and urinary ACR, and reduced eGFR), higher LAP, WC, weight, BMI, TG, blood pressure (SBP, DBP) and glucose (FBG, PBG, and HbA1c), prevalence of obesity, hypertension, T2DM, CVD events (CHD and PAD), and lower HDL-C compared with those without (*P* < 0.001 or *P* < 0.01 or *P* < 0.05; Table 1 and Fig. 2). Further analysis indicated that only female but not male subjects with albuminuria and low eGFR displayed higher levels of LAP than the corresponding controls (all *P* < 0.001, Fig. 2).

**Table 1:** Clinical and biochemical characteristics of study population

	Men			Women		
	non-CKD (*n* = 2011)	CKD (*n* = 302)	***P***	non-CKD (*n* = 4256)	CKD (*n* = 633)	***P***
Age (years)	59.00 (53.00–66.00)	64.00 (56.00–72.00)	< 0.001	57.00 (54.60–64.90)	61.00 (54.00–70.00)	< 0.001
WC (cm)	85.30 ± 9.85	86.24 ± 9.51	0.120	81.00 (75.00–87.20)	83.00 (77.00–90.00)	< 0.001
LAP	25.41 (13.58–45.72)	28.12 (16.30–54.49)	0.117	29.16 (17.00–49.30)	38.74 (21.75–67.42)	< 0.001
Height (cm)	164.10 (160.00–168.90)	163.20 (159.00–168.00)	0.027	154.10 (150.20–158.00)	154.00 (150.00–158.00)	0.081
Weight (kg)	64.00 (57.50–71.20)	64.00 (58.00–71.00)	0.970	56.00 (50.90–62.00)	56.50 (51.50–63.00)	0.014
BMI (kg/m^2^)	23.80 (21.63–26.03)	24.02 (22.03–25.99)	0.173	23.53 (21.43–25.86)	24.19 (21.78–26.55)	< 0.001
SBP (mmHg)	124.33 (112.00–138.67)	135.00 (120.00–149.00)	< 0.001	120.00 (107.67–134.00)	128.67 (114.33–145.50)	< 0.001
DBP (mmHg)	77.67 (70.67–85.00)	79.00 (71.00–90.00)	0.102	74.00 (67.67–80.67)	76.67 (69.33–85.00)	< 0.001
TC (mmol/L)	4.51 ± 1.08	4.69 ± 1.09	0.010	4.82 ± 1.10	4.90 ± 1.21	0.117
TG (mmol/L)	1.28 (0.90–1.87)	1.35 (0.95–2.08)	0.132	1.29 (0.91–1.88)	1.52 (1.07–2.19)	< 0.001
HDL-C (mmol/L)	1.17 (0.97–1.38)	1.19 (0.99–1.36)	0.576	1.32 (1.10–1.56)	1.26 (1.04–1.49)	< 0.001
LDL-C (mmol/L)	2.52 (2.00–3.09)	2.70 (2.14–3.23)	0.007	2.66 (2.12–3.23)	2.66 (2.12–3.32)	0.825
FBG (mmol/L)	5.49 (5.13–6.12)	5.63 (5.21–6.70)	0.001	5.39 (5.07–5.89)	5.53 (5.18–6.31)	< 0.001
PBG (mmol/L)	7.92 (6.37–10.70)	8.70 (6.88–12.72)	< 0.001	7.51 (6.26–9.83)	8.58 (6.87–11.99)	< 0.001
HbA1c (%)	5.90 (5.60–6.30)	6.00 (5.70–6.60)	0.001	5.90 (5.60–6.30)	6.00 (5.60–6.50)	< 0.001
Cr (μmol/L)	71.90 (65.40–81.30)	78.75 (68.88–106.20)	< 0.001	59.80 (54.60–64.90)	63.20 (55.35–81.65)	< 0.001
eGFR (mL/min/1.73 m^2^)	96.65 (88.30–103.89)	88.83 (59.23–100.20)	< 0.001	97.17 (89.16–104.19)	90.72 (63.51–100.66)	< 0.001
Urinary ACR (mg/g)	7.74 (5.08–12.30)	39.30 (31.54–56.69)	< 0.001	6.61 (4.32–11.16)	43.75 (31.79–61.89)	< 0.001
Current smoker (%)	794 (39.48%)	106 (35.10%)	0.145	65 (1.53%)	11 (1.74%)	0.690
Current drinkers (%)	1085 (53.95%)	144 (47.68%)	0.042	643 (15.11%)	79 (12.48%)	0.082
Overweight (%)	730 (36.30%)	119 (39.40%)	0.297	1460 (34.30%)	232 (36.65%)	0.247
Obesity (%)	223 (11.09%)	33 (10.93%)	0.933	448 (10.53%)	100 (15.80%)	< 0.001
Prediabetes (%)	581 (28.89%)	76 (25.17%)	0.181	1163 (27.33%)	190 (30.02%)	0.158
T2DM (%)	537 (26.70%)	113 (37.42%)	< 0.001	854 (20.07%)	210 (33.18%)	< 0.001
Users of hypoglycemic drugs (%)	195 (9.70%)	46 (15.23%)	0.003	299 (7.03%)	84 (13.27%)	< 0.001
Hypertension (%)	726 (36.10%)	163 (53.97%)	< 0.001	1117 (26.25%)	316 (49.92%)	< 0.001
MI (%)	13 (0.65%)	5 (1.66%)	0.063	8 (0.19%)	3 (0.47%)	0.157
CHD (%)	68 (3.38%)	18 (5.96%)	0.030	107 (2.51%)	39 (6.16%)	< 0.001
Stroke (%)	15 (0.75%)	4 (1.32%)	0.299	16 (0.38%)	3 (0.47%)	0.712
PAD (%)	1 (0.05%)	3 (0.99%)	< 0.001	2 (0.05%)	2 (0.32%)	0.027
CVD (%)	88 (4.38%)	26 (8.61%)	0.002	129 (3.03%)	45 (7.11%)	< 0.001

Data are mean ± SD. *SD* Standard deviation, *WC* Waist circumference, *LAP* Lipid accumulation product, *BMI* Body mass index, *SBP* Systolic blood pressure, *DBP* Diastolic blood pressure, *TC* Total cholesterol, *TG* Triglyceride, *HDL-C* High-density lipoprotein cholesterol, *LDL-C* Low-density lipoprotein cholesterol, *FBG* Fasting blood glucose, *PBG* 2 h postload blood glucose, *HbA1c* Glycated hemoglobin A1c, *Cr* Creatinine, *eGFR* Estimated glomerular filtration rate, *ACR* Albumin-to-creatinine ratio, *T2DM*, type 2 diabetes mellitus, *MI* Myocardial infarction, *CHD* Coronary heart disease, *PAD* Peripheral arterial disease, *CVD* Cardiovascular disease

### Univariate analysis of variables contributing to CKD prevalence

The prevalence of CKD showed a significantly positive correlation with age, blood pressure, TC, LDL-C, serum Cr, urinary ACR, prevalence of T2DM and CVD events, and use of hypoglycaemic drugs, but the correlation between CKD prevalence and eGFR was negative in male subjects (*P* < 0.001 or *P* < 0.01 or *P* < 0.05; Table 2). In female subjects, the prevalence of CKD was significantly and positively associated with age, BMI, WC, LAP, blood pressure, TG, serum Cr, urinary ACR,prevalence of T2DM and CVD events, use of hypoglycaemic drugs, and negatively with HDL-C and eGFR (*P* < 0.001 or *P* < 0.01 or *P* < 0.05; Table 2).

**Table 2:** Univariate analysis of variables contributing to CKD in study population

	Men			Women		
	Univariate analysis					
	B	OR (95%CI)	***P***-value	B	OR (95%CI)	***P***-value
Age	0.039	1.040 (1.027–1.053)	< 0.001	0.046	1.047 (1.038–1.056)	< 0.001
BMI	0.020	1.020 (0.989–1.053)	0.206	0.031	1.032 (1.014–1.051)	0.001
WC	0.010	1.010 (0.998–1.022)	0.120	0.024	1.024 (1.016–1.033)	< 0.001
LAP	0.002	1.002 (0.999–1.004)	0.119	0.006	1.006 (1.005–1.008)	< 0.001
SBP	0.020	1.020 (1.014–1.027)	< 0.001	0.022	1.022 (1.018–1.026)	< 0.001
DBP	0.013	1.013 (1.003–1.024)	0.015	0.027	1.027 (1.019–1.035)	< 0.001
TC	0.144	1.155 (1.035–1.290)	0.010	0.064	1.066 (0.990–1.148)	0.092
TG	0.041	1.041 (0.964–1.125)	0.302	0.183	1.201 (1.135–1.270)	< 0.001
HDL-C	0.093	1.098 (0.756–1.595)	0.624	−0.481	0.618 (0.482–0.792)	< 0.001
LDL-C	0.194	1.215 (1.050–1.405)	0.009	0.013	1.013 (0.916–1.120)	0.800
Cr	0.046	1.047 (1.039–1.055)	< 0.001	0.056	1.058 (1.050–1.065)	< 0.001
eGFR	−0.050	0.951 (0.943–0.958)	< 0.001	− 0.050	0.951 (0.946–0.956)	< 0.001
Urinary ACR	0.193	1.213 (1.189–1.237)	< 0.001	0.186	1.204 (1.188–1.220)	< 0.001
Drinking	−0.201	0.818 (0.638–1.048)	0.111	−0.214	0.808 (0.629–1.037)	0.094
Smoking	−0.110	0.896 (0.695–1.155)	0.396	0.323	1.381 (0.758–2.514)	0.291
T2DM	0.495	1.641 (1.274–2.114)	< 0.001	0.682	1.978 (1.649–2.372)	< 0.001
Hypoglycemic drugs	0.515	1.673 (1.183–2.367)	0.004	0.706	2.205 (1.564–2.621)	< 0.001
CVD events	0.722	2.059 (1.306–3.246)	0.002	0.895	2.448 (1.725–3.475)	< 0.001

Beta is the standardized coefficient and measures the influence of each variable on CKD; OR is the odds ratio and refers to the risk of CKD

### Association of LAP quartiles with the risk of CKD prevalence

Table 3 revealed the correlation of LAP quartiles with the risk of CKD prevalence in men and women, respectively. The results revealed that this association of LAP quartiles with the risk of CKD prevalence in men was weakened and lost significance after adjustment for age, BMI, smoking, drinking, prevalence of CVD events, blood pressure, hypoglycemic drugs, prevalence of T2DM, and LDL-C in Model 4 (*P* for trend > 0.05). In women, the risk of CKD prevalence increased by 28.2% (95% CI 19.8–37.2%; *P* < 0.001) per SD increase in LAP levels, and this association was weakened but remained significant after adjustment for multiple variables in Model 2–4 (all *P* < 0.001). Consistently, the risk of CKD prevalence progressively increased across LAP quartiles (*P* for trend < 0.001 or *P* for trend < 0.01), and the risk of CKD prevalence of subjects in Q4 significantly increased compared to those in Q1 in Models 1–4 (*P* < 0.001 or *P* < 0.01 or *P* < 0.05).

**Table 3:** Association between LAP quartiles and risk of CKD in the study population

Variable	Per SD increase	LAP quartiles				***P*** for trend
		Q1	Q2	Q3	Q4	
**Men**						
Model 1						
OR (95%CI)	1.088 (0.978–1.210)	1	1.228 (0.860–1.753)	1.193 (0.846–1.682)	1.570 (1.131–2.178)	0.029
*P*-value	0.119		0.258	0.315	0.007	
Model 2						
OR (95%CI)	1.116 (0.991–1.256)	1	1.215 (0.819–1.803)	1.173 (0.793–1.735)	1.725 (1.115–2.668)	0.040
*P*-value	0.069		0.332	0.423	0.014	
Model 3						
OR (95%CI)	1.118 (0.993–1.260)	1	1.210 (0.814–1.799)	1.173 (0.790–1.741)	1.725 (1.110–2.678)	0.037
*P*-value	0.066		0.346	0.430	0.015	
Model 4						
OR (95%CI)	1.081 (0.951–1.228)	1	1.069 (0.705–1.621)	1.003 (0.651–1.544)	1.393 (0.870–2.228)	0.320
*P*-value	0.232		0.754	0.991	0.167	
**Women**						
Model 1						
OR (95%CI)	1.282 (1.198–1.372)	1	1.096 (0.839–1.431)	1.459 (1.132–1.881)	2.173 (1.696–2.784)	< 0.001
*P*-value	< 0.001		0.501	0.003	< 0.001	
Model 2						
OR (95%CI)	1.224 (1.137–1.318)	1	0.979 (0.738–1.299)	1.157 (0.869–1.540)	1.599 (1.190–2.148)	< 0.001
*P*-value	< 0.001		0.883	0.318	0.002	
Model 3						
OR (95%CI)	1.220 (1.133–1.313)	1	0.988 (0.744–1.311)	1.132 (0.848–1.510)	1.582 (1.176–2.127)	< 0.001
*P*-value	< 0.001		0.931	0.400	0.002	
Model 4						
OR (95%CI)	1.169 (1.082–1.262)	1	0.981 (0.730–1.317)	1.051 (0.773–1.430)	1.382 (1.002–1.906)	0.002
*P*-value	< 0.001		0.898	0.751	0.048	

Data are expressed as OR (95% CI) + *P* value, unless stated otherwise. Model 1: unadjusted; Model 2: adjusted for age and BMI; Model 3: additionally adjusted for smoking, drinking, prevalence of CVD events based on Model 2; Model 4: additionally adjusted for SBP, DBP, hypoglycemic drugs, LDL-C, and prevalence of T2DM based on Model 3

### Association of LAP quartiles with risk CKD prevalence in a stratified analysis

The results showed that the associations of higher LAP quartiles with an increased risk of CKD prevalence were not consistently the same (Table 4). A significant correlation between higher LAP quartiles and an elevated risk of CKD prevalence was only detected in subjects who were women, aged ≥60 years, overweight, having NGT, hypertension, appropriate LDL-C, and no smoking, drinking, and CVD events (*P* < 0.01 or *P* < 0.05). Furthermore, an interaction of blood pressure stratification with LAP quartiles was found in CKD (*P* for interaction < 0.001).

**Table 4:** Association between LAP quartiles and increased risk of CKD in different participants

Variable	LAP quartiles				***P*** for trend	***P*** for interaction
	Q1	Q2	Q3	Q4		
	OR (95%C) ***P***-value	OR (95%CI) ***P***-value	OR (95%CI) ***P***-value	OR (95%CI) ***P***-value		
Gender						0.511
Men	1	1.269 (0.851–1.892) 0.243	0.912 (0.584–1.425) 0.687	1.472 (0.905–2.394) 0.120	0.211	
Women	1	0.982 (0.721–1.337) 0.908	0.989 (0.715–1.368) 0.947	1.462 (1.041–2.054) 0.029	0.006	
Age, years						0.061
< 60	1	1.098 (0.784–1.539) 0.586	1.077 (0.737–1.572) 0.702	1.622 (1.086–2.425) 0.018	0.050	
≥ 60	1	1.071 (0.751–1.527) 0.705	0.927 (0.641–1.340) 0.686	1.427 (0.970–2.100) 0.071	0.014	
BMI status						0.064
Normal	1	1.096 (0.828–1.451) 0.522	0.914 (0.648–1.289) 0.609	1.288 (0.861–1.926) 0.218	0.492	
Overweight	1	1.110 (0.860–1.434) 0.422	0.993 (0.743–1.328) 0.963	1.473 (1.072–2.024) 0.017	0.002	
Obesity	1	1.510 (0.099–23.014) 0.767	4.444 (0.418–47.272) 0.216	2.061 (0.240–17.699) 0.510	0.125	
Glucose metabolism status						0.636
NGT	1	0.868 (0.624–1.208) 0.401	0.923 (0.633–1.345) 0.676	1.697 (1.117–2.577) 0.013	0.019	
Prediabetes	1	1.495 (0.904–2.472) 0.117	1.106 (0.650–1.884) 0.710	1.673 (0.943–2.969) 0.078	0.592	
T2DM	1	1.179 (0.662–2.101) 0.576	0.855 (0.492–1.485) 0.577	1.379 (0.798–2.381) 0.249	0.060	
Blood pressure status						< 0.001
Normal	1	1.061 (0.793–1.421) 0.689	0.932 (0.672–1.294) 0.675	1.568 (1.088–2.259) 0.016	0.065	
Hypertension	1	1.122 (0.717–1.756) 0.615	1.056 (0.667–1.671) 0.816	1.427 (0.908–2.242) 0.123	0.035	
LDL-C						0.255
Appropriate	1	1.177 (0.906–1.529) 0.222	1.052 (0.790–1.402) 0.727	1.505 (1.116–2.028) 0.007	0.014	
Borderline high	1	0.622 (0.265–1.461) 0.276	0.788 (0.354–1.753) 0.559	1.908 (0.787–4.622) 0.153	0.059	
High	1	0.326 (0.078–1.356) 0.123	0.207 (0.050–0.859) 0.030	0.891 (0.256–3.095) 0.856	0.747	
CVD events						0.653
No	1	1.066 (0.832–1.365) 0.613	0.920 (0.705–1.201) 0.539	1.476 (1.119–1.948) 0.006	0.007	
Yes	1	3.388 (0.685–16.748) 0.134	2.337 (0.683–8.005) 0.176	2.236 (0.447–11.198) 0.328	0.166	
Current drinking status						0.640
No	1	1.010 (0.757–1.350) 0.944	0.910 (0.671–1.234) 0.543	1.450 (1.051–2.000) 0.024	0.004	
Yes	1	1.238 (0.786–1.950) 0.357	1.070 (0.637–1.798) 0.799	1.471 (0.858–2.523) 0.161	0.590	
Current smoking status						0.413
No	1	1.082 (0.831–1.407) 0.559	1.019 (0.768–1.353) 0.895	1.455 (1.083–1.954) 0.013	0.004	
Yes	1	1.011 (0.525–1.946) 0.974	0.705 (0.355–1.400) 0.318	1.847 (0.848–4.026) 0.122	0.494	

Data are expressed as OR (95% CI) + *P* value, unless stated otherwise. Adjusted for sex, age, BMI, smoking, drinking, CVD events, SBP, DBP, LDL-C, prevalence of T2DM, and hypoglycemic drugs

## Discussion

This is currently the first study to show an increased levels of LAP in community-dwelling Chinese female not male adults with CKD, defined as low eGFR and/or the presence of albuminuria. The multivariate logistic regression analysis revealed that the risk of CKD prevalence in female not male subjects progressively increased across LAP quartiles, and the risk of CKD prevalence of subjects in Q4 significantly increased compared to those in Q1 after adjustment for potential confounding factors. Additionally, stratified analysis showed that subjects with higher LAP quartiles had an increased risk of CKD prevalence than those with lower quartiles, especially in women, people who were older, overweight, with hypertension, NGT, appropriate LDL-C, and without smoking, drinking, and CVD events.

There is evidence to suggest that obesity, especially central obesity, is an important risk factor for CKD [6–9]. LAP, an available and emerging surrogate marker of central obesity, can be used to assess the visceral adiposity distribution and reflect visceral adiposity dysfunction [11, 12], and thus, it might be involved in the development and progression of CKD. Previous cross-sectional studies regarding the relationship of LAP with CKD (simply defined by low eGFR but not evaluated by albuminuria) have yielded conflicting findings [11, 13, 14]. An observational study in East China demonstrated that in 10,012 male and female subjects aged ≥18 years, LAP levels displayed a strong association with low eGFR and could predict the risk of renal dysfunction [13]. Another study of 11,192 individuals aged ≥35 years also reported a significant association of LAP with low eGFR, and LAP could predict low eGFR only in women in rural population of northeast China [14]. Seong and colleagues has recently demonstrated that LAP quartiles were association with the prevalence of low eGFR in 4947 Korean subjects (aged ≥20 years); however, the association was observed only in men after adjusting for related variables [11]. The present study was consistent with those reports of Zhang et al. and Dai et al. [13, 14], but was inconsistent with the findings reported by Seong et al. [11], altogether suggesting that high LAP might be associated with CKD prevalence. These discrepancies between the current study and previous cross-sectional studies might be due to differences in population characteristics such as age and male/female proportion, study groups, region, races, dietary habits, CKD definition, sample sizes, and confounding factors adjusted in the analyses. Surprisingly, the results from a prospective study, consisting of 6693 Iranian non-diabetic adults aged ≥18 years, revealed that eGFR progressively decreased across LAP tertiles at baseline, but LAP was not a significant predictor of incident renal function decline in either sex after adjusting for multiple confounders [15], which was inconsistent with the present study. These discrepancies between the two studies might be due to differences in population characteristics such as age and male/female proportion, study groups, study designs, CKD definition, races, dietary habits, statistical methods, and confounding factors adjusted in the analyses. Additional studies are needed to confirm the present findings.

Interestingly, the present study revealed a significant sex difference in the effect of LAP on CKD prevalence because female but not male subjects with CKD, albuminuria, and low eGFR had significantly higher LAP levels compared with the respective control groups. Moreover, the logistic regression analysis revealed that LAP level of female not male subjects were significantly and positively associated with CKD prevalence, and the risk of CKD prevalence in female not male subjects progressively increased across LAP quartiles after adjustment for potential confounding factors. Further stratification by sex revealed that a significant correlation between higher LAP quartiles and risk of CKD prevalence was only observed in women, whereas such a relationship was not detected in men after multivariable adjustment, consistent with a previous study [14]. These findings suggest a sex-specific association of LAP with CKD prevalence, the underlying mechanisms of which might be attributed to differences in sex hormones, fat distribution, and lifestyles between men and women. The vast majority of women in the current study were > 50 years of age and thus presumably in the perimenopausal or postmenopausal stage with significantly decreased production of oestrogen, which would lead to adipose tissue redistribution characterized by more abdominal VAT, secreting various bioactive adipocytokines and inflammatory proteins, and contributing to changes in renal haemodynamics, renal vascular damage, glomerular sclerosis, renal fibrosis, low eGFR, albuminuria, and CKD [7, 22, 23]. Conversely, male subjects tended to be physically active and to consume more green tea, both of which may lead to a lower distribution of abdominal fat in males compared with females at the same age who were less active and consumed less tea [24–26]. Several reports have suggested that age is an important risk factor for CKD [11]. The present study also revealed that the prevalence of CKD was significantly and positively associated with age, and also suggested a positive relationship of LAP quartiles with risk of CKD prevalence in subjects aged ≥60 years but not those aged < 60 years, which was similar to a previous study [27]. These results suggest older age might be associated with CKD prevalence because elderly individuals have higher prevalenceof comorbidities, such as obesity, hyperuricaemia, dyslipidaemia, diabetes, and hypertension, and more use of medications for these chronic diseases, which may have a profound negative impact on kidney function. Overweight/obesity has been reported to be involved in the development of CKD [5–8]. The present study also showed that female subjects with CKD had significantly higher BMI and prevalence of obesity compared with those without CKD, and BMI was positively correlated with CKD prevalence. Further stratified analysis by BMI revealed that a significant correlation between higher LAP quartiles and risk of CKD prevalence was only detected in overweight subjects, which was similar to a previous study [28], suggesting that being overweight may correlate with the prevalence of CKD via several modifiable atherosclerotic and cardiometabolic risks, as well as endothelial dysfunction. Moreover, after adjusting for sex, age, and BMI, the correlation of LAP quartiles with risk of CKD prevalence in female subjects was attenuated, indicating that female sex, older age, and overweight status added to the risk of CKD prevalence and altogether probably weakened the correlation of LAP quartiles with risk of CKD prevalence. Thus, it is of great importance to monitor LAP for early screening for CKD among people who were women, older, and overweight to further prevent and delay the development and progression of CKD by incorporating changes in lifestyle and necessary medical treatments.

Emerging epidemiologic studies have suggested that diabetes, hypertension, and dyslipidaemia are important risk factors for CKD [1, 4, 6, 27–29]. Thepresent study also showed that male and female subjects with CKD had significantly more users of hypoglycaemic drugs, higher blood glucose (FBG, PBG, and HbA1c), prevalence of T2DM and hypertension compared with those without CKD. Moreover, the prevalence of CKD showed a significantly positive correlation with blood pressure, prevalence of T2DM, and use of hypoglycaemic drugs in both sexes. These results suggested that the prevalence of CKD might be correlated with the prevalence of diabetes and hypertension. Additionally, the correlation of LAP quartiles with CKD prevalence in female subjects was attenuated but retained significance after further adjusting for blood pressure, LDL-C, prevalence of T2DM, and hypoglycaemic drugs in Model 4, suggesting that diabetes, hypertension, and dyslipidaemia added to the risk of CKD prevalence and probably weakened the correlation of LAP with of risk of CKD prevalence. Further stratified analysis showed that a significant correlation of higher LAP quartiles with an increased risk for CKD prevalence was only detected in hypertensive subjects but not in those with normal blood pressure, which was generally in line with several previous studies [28, 30, 31], suggesting that hypertension might be associated with the prevalence of CKD. More importantly, an interaction of hypertension with LAP on CKD prevalence was also observed, indicating that hypertension and elevated LAP may synergistically increase the risk of CKD prevalence, and hypertension might a stronger factor affecting the relationship between LAP and CKD prevalence. It is suggested that the most important lipid risk factor and therapeutic target for CVD is LDL-C [32]; however, there is still a considerable residual risk of CVD events even after a reduction of LDL-C to the recommended concentrations with statins and other lipid-lowering agents [31]. It is generally accepted that CKD and CVD share common risk factors, such as diabetes mellitus and dyslipidaemia [33, 34]. Of note, stratified analysis also revealed that significant associations between higher LAP quartiles and CKD were still detected in people with NGT and appropriate LDL-C (< 3.4 mmol/L) as recommended by the guidelines, indicative of an excess risk in people with NGT and appropriate LDL-C. Additionally, the risk of CKD might already be present in community-dwelling Chinese adults with NGT and appropriate LDL-C. The lack of significant associations of LAP with CKD prevalence among subjects with prediabetes, T2DM, and abnormal LDL-C is speculated to be due to the following factors: first, subjects with prediabetes, T2DM, abnormal LDL-C might be inclined to change their diet and lifestyle habits and receive related treatment, thus contributing to the favourable effect of CKD prevention. Additionally, they were individually and jointly related to a decreased risk of CKD. Second, both experimental and clinical data have identified T2DM and dyslipidaemia as important causes of CKD, so it is reasonable to speculate that the strong relationships of prediabetes, T2DM, and abnormal LDL-C with CKD probably weakens the correlation of LAP with risk of CKD prevalence. Third, the small sample size of some subgroups, especially borderline high LDL-C (*n* = 973) and high LDL-C (*n* = 355), and the large 95% CIs have resulted in data availability or imprecise estimations. Further studies with larger sample sizes and detailed information about dietary structures, lifestyle habits, and medications are needed to investigate the association of LAP with CKD in different subgroups.

Many studies have reported significant relationships of smoking, drinking, and CVD with CKD [4, 34, 35,36]. The present study showed that patients with CKD had significantly higher prevalence of CVD events (CHD and PAD) than those with non-CKD, suggesting that the prevalence of CKD might be correlated with CVD events. Moreover, a significantly positive correlation between prevalence of CKD and CVD events. Additionally, after further adjusting for smoking, drinking, and prevalence of CVD events, the correlation of LAP quartiles with risk of CKD prevalence in female subjects was attenuated but retained significance, suggesting that smoking, drinking, and CVD added to the risk of CKD prevalence and probably weakened the correlation of LAP with risk of CKD prevalence. However, the stratified analysis demonstrated a significant correlation of higher LAP quartiles with an increased risk for CKD prevalence in subjects without smoking, drinking, and CVD events but not those with, which be due to several factors. First, both experimental and clinical data have identified smoking, drinking, and CVD as important causes of CKD, so it is reasonable to speculate that the strong relationships of smoking, drinking and CVD events with CKD likely weakened the correlation of LAP with risk of CKD prevalence. Second, the small sample sizes of some subgroups, especially CVD events (*n* = 288) and smokers (*n* = 976) and the large 95% CIs led to data availability or imprecise estimations. Further research in a larger population is needed to verify our speculation.

### Study strengths and limitations

Several potential limitations exist in the present study. First, the cross-sectional study design precluded the establishment of causality, which requires confirmation in large-scale longitudinal studies. Second, although those subjects who used ACEI/ARB and lipid-lowering drugs were excluded and hypoglycaemic drugs were adjusted, it is still possible that other drugs could partially affect the correlation between LAP and CKD prevalence. Third, the population in the present study included only community-dwelling middle-aged and elderly subjects in Luzhou city located within Sichuan Province in Southwest China; therefore, the present findings may not be generalizable to other populations. Despite these limitations, the current study is not without strengths, including a standardized method of data collection, comprehensive adjustment for major traditional risk factors, and a relatively large sample size, which can strengthen the reliability of our findings. More importantly, the present study is the first to provide clinical evidence for a potential link between LAP and CKD prevalence, defined as low eGFR and/or presence of albuminuria, in Chinese community adults.

## Conclusions

Higher LAP might be significantly related to risk of CKD prevalence in Chinese community female adults. Subjects with elevated LAP had a higher risk for CKD prevalence, especially those who were older, overweight, with hypertension, NGT, appropriate LDL-C, and without smoking, drinking, and CVD events. These results indicate that LAP may be a useful and reliable indicator of CKD prevalence and highlight the importance of paying more attention to CKD in middle-aged and elderly subjects with central obesity to further reduce CKD-associated unfavourable health outcomes.

## Abbreviations

CKD: Chronic kidney disease
CVD: Cardiovascular disease
BMI: Body mass index
WC: Waist circumference
LAP: Lipid accumulation product
TG: Triglyceride
eGFR: Estimated glomerular filtration rate
ACR: Albumin-to-creatinine ratio
Q: Quartile
SBP: Systolic blood pressure
DBP: Diastolic blood pressure
FBG: Fasting blood glucose
PBG: 2 h postload blood glucose
HbA1c: Glycated hemoglobin A1c
TC: Total cholesterol
HDL-C: High-density lipoprotein cholesterol
LDL-C: Low-density lipoprotein cholesterol
Cr: Creatinine
CKD-EPI: Chronic Kidney Disease Epidemiology Collaboration
T2DM: Type 2 diabetes mellitus
NGT: Normal glucose tolerance
MI: Myocardial infarction
CHD: Coronary heart disease
PAD: Peripheral arterial disease
SD: Standard deviation
OR: Odds ratios
CI: Confidence intervals
CT: Computed tomography
MRI: Magnetic resonance imaging
VAT: Visceral adipose tissue
SAT: Subcutaneous adipose tissue
REACTION: Risk Evaluation of Cancers in Chinese Diabetic Individuals
ACEI: Angiotensin-converting enzyme inhibitor
ARB: Angiotensin receptor blocker
ISO: International Organization for Standardization
